# Consumer Perspectives of Safewards Impact in Acute Inpatient Mental Health Wards in Victoria, Australia

**DOI:** 10.3389/fpsyt.2019.00461

**Published:** 2019-07-09

**Authors:** Justine Fletcher, Sally Buchanan-Hagen, Lisa Brophy, Stuart A. Kinner, Bridget Hamilton

**Affiliations:** ^1^Centre for Mental Health, Melbourne School of Population and Global Health, The University of Melbourne, Melbourne, VIC, Australia; ^2^School of Nursing and Midwifery, Deakin University, Geelong, VIC, Australia; ^3^School of Allied Health, Human Services and Sport, La Trobe University, Melbourne, VIC, Australia; ^4^Mind Australia Limited, Heidelberg, VIC, Australia; ^5^Melbourne School of Population and Global Health, The University of Melbourne, Carlton, VIC, Australia; ^6^Centre for Adolescent Health, Murdoch Children’s Research Institute, Parkville, VIC, Australia; ^7^Mater Research Institute-UQ, University of Queensland, Brisbane, QLD, Australia; ^8^Griffith Criminology Institute, Griffith University, Mt Gravatt, QLD, Australia; ^9^Centre for Psychiatric Nursing, School of Health Sciences, The University of Melbourne, Melbourne, VIC, Australia

**Keywords:** inpatient, Safewards, seclusion, wards, restrictive interventions, consumer perspective, service users

## Abstract

**Background:** Inpatient mental health wards are reported by many consumers to be custodial, unsafe, and lacking in therapeutic relationships. These consumer experiences are concerning, given international policy directives requiring recovery-oriented practice. Safewards is both a model and a suite of interventions designed to improve safety for consumers and staff. Positive results in reducing seclusion have been reported. However, the voice of consumers has been absent from the literature regarding Safewards in practice.

**Aim:** To describe the impact of Safewards on consumer experiences of inpatient mental health services.

**Method:** A postintervention survey was conducted with 72 consumers in 10 inpatient mental health wards 9–12 months after Safewards was implemented.

**Results:** Quantitative data showed that participants felt more positive about their experience of an inpatient unit, safer, and more connected with nursing staff. Participants reported that the impact of verbal and physical aggression had reduced because of Safewards. Qualitatively, participants reported increased respect, hope, sense of community, and safety and reduced feelings of isolation. Some participants raised concerns about the language and intention of some interventions being condescending.

**Discussion:** Consumers’ responses to Safewards were positive, highlighting numerous improvements of importance to consumers since its implementation across a range of ward types. The findings suggest that Safewards offers a pathway to reducing restrictive interventions and enables a move toward recovery-oriented practice.

## Introduction

In the contemporary Australian mental health service system, acute inpatient wards are challenging settings where a high proportion of consumers are involuntarily admitted, for example, in the years 2016–2017, 57% of Victorian inpatient admissions were involuntary (1). In this paper, we use the term “consumer” to describe people who experience mental distress and use public mental health services because this is the most commonly used term in Australia. Consumers of inpatient wards are vulnerable and in need of skilled and empathic care. Unfortunately, internationally, consumers report a myriad of harmful experiences during their inpatient care (2), many associated with restrictive practices. Such harms may be a contributing factor to suicides both during and after admission (3, 4). A long-standing imperative within service systems is to improve the consumer experience in inpatient wards, including decreasing harms (5, 6).

Previous research that has involved consumers providing feedback about their experience of inpatient services has identified a multitude of challenges to providing services that meet consumers’ expectations for care and treatment. Consumers report that inpatient wards are custodial (7) and sterile (8), with stringent and arbitrary rules (9) and lacking fairness and respect for consumers (2). Consumers report feeling bored, in need of distraction (8), and unsafe (10, 11) and that staff do not have time for therapeutic engagement (8, 10).

With such challenges comes tension between staff and consumers and sometimes between consumers and other consumers (12, 13). These tensions can lead to conflict, such as aggression, substance use, or absconding (14), which can then result in the use of restrictive practices, sometimes described as containment (14). Containment practices, such as seclusion and restraint, and the use of force have negative consequences for consumers who experience them and for those who witness them (7, 9, 10). Criticisms of restrictive practices have been further highlighted in the UN Convention on the Rights of Persons with Disability (15).

Safewards is a model and set of 10 interventions designed to improve safety for both consumers and staff in inpatient wards by reducing conflict and containment (16), attracting wide interest as an intervention that can reduce the use of restrictive practices.

**Figure 1:**
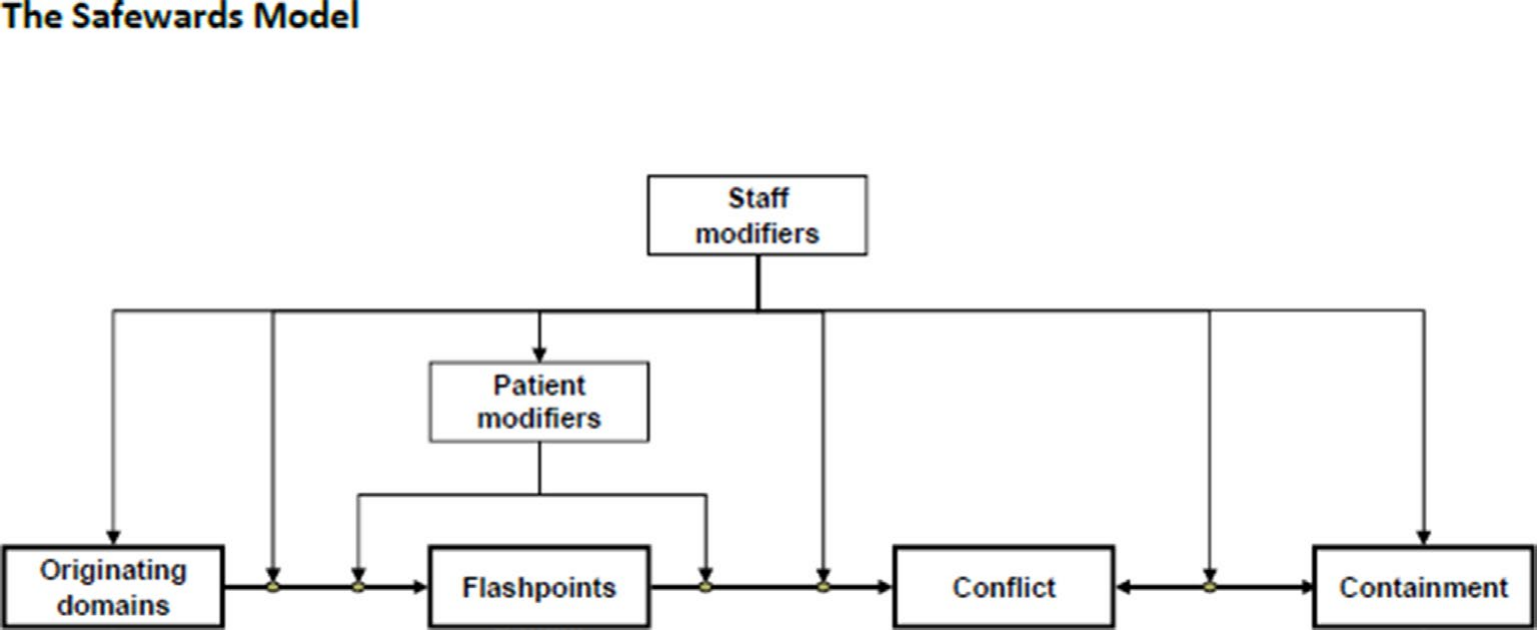
Simple form Safewards Model (16, p. 500).

According to the Safewards model, multiple factors influence conflict and containment events in acute mental health inpatient settings. The model suggests a linear relationship such that originating domains precipitate a flashpoint that can then set in motion an incident of conflict possibly resulting in containment. The relationship between conflict and containment is reciprocal in that the use of containment can lead to further conflict (16) (see **Figure 1** for the model and **Box 1** for definitions of the model components). The model also suggests that the influence of staff modifiers is present at every level. Patient modifiers can influence processes either before or after a flashpoint, and patient modifiers are also influenced by staff modifiers (16).

Box 1 **Defining the components of the Safewards model (16).**

**Originating domains**: six categories describing aspects of psychiatric wards: *patient community, patient characteristics, regulatory framework, staff team, physical environment*, and *outside hospital*. The frequencies of conflict and containment are influenced by the degree to which each of these originating domains is present or absent.

**Staff modifiers**: relates to staff as individuals or the team and the capacity they have to influence conflict and containment, by how they act to manage patients and the ward environment, initiating or responding to interactions with patients.

**Patient modifiers**: the way patients respond and behave toward each other that can influence the frequency of conflict and containment. Staff can also influence these.

**Flashpoints**: influenced by the originating domains, these are social and psychological situations, signaling and preceding imminent conflict behaviors.

**Conflict**: patient behaviors that threaten safety or the safety of others (e.g., violence, suicide, self-harm, absconding).

**Containment**: things staff do to prevent conflict from occurring or minimize harmful outcomes (e.g., prn medication, special observation, seclusion, restraint).

More than 30 interventions were developed by the research team in the original design; consultation with expert nurses, consumers, and carer representatives from SUGAR (Service User and Carer Group Advising on Research) narrowed the pool of interventions to 16, which were then piloted in 2012. Subsequently, 10 interventions were included in a randomized controlled trial (RCT). The 10 interventions can be categorized into two groups (described in **Table 1**). The first group (noted with a 1 in brackets) included interventions that actively involve consumers in collaboration with staff in the ward. The second group of interventions (noted with a 2 in brackets) requires active change of clinicians’ practice to implement new ways of working.

**Table 1:** Safewards Interventions.

Intervention	Description	Purpose
**Mutual Help Meeting (1)**	Patients offer and receive mutual help and support through a daily, shared meeting.	Strengthens patient community, opportunity to give and receive help
**Know Each Other (1)**	Patients and staff share some personal interests and ideas with each other, displayed in unit common areas.	Builds rapport, connection, and sense of common humanity
**Clear Mutual Expectations (1)**	Patients and staff work together to create mutually agreed aspirations that apply to both groups equally.	Counters some power imbalances, creates a stronger sense of shared community
**Calm Down Methods (1)**	Staff support patients to draw on their strengths and use/learn coping skills before the use of PRN medication or containment.	Strengthen patient confidence and skills to cope with distress
**Discharge Messages (1)**	Before discharge, patients leave messages of hope for other patients on a display in the unit.	Strengthens patient community, generates hope
**Soft Words (2)**	Staff take great care with their tone and use of collaborative language. Staff reduce the limits faced by patients, create flexible options, and use respect if limit setting is unavoidable.	Reduces a common flashpoint Builds respect, choice, and dignity
**Talk Down (2)**	De-escalation process focuses on clarifying issues and finding solutions together. Staff maintain self-control, respect, and empathy.	Increases respect, collaboration and mutually positive outcomes
**Positive Words (2)**	Staff say something positive in handover about each patient. Staff use psychological explanations to describe challenging actions.	Increases positive appreciation and helpful information for colleagues to work with patients
**Bad News Mitigation (2)**	Staff understand, proactively plan for, and mitigate the effects of bad news received by patients.	Reduces impact of common flashpoints, offers extra support
**Reassurance (2)**	Staff touch base with every patient after every conflict on the unit and debrief as required.	Reduces a common flashpoint, increases patients’ sense of safety and security

Adapted from the DHHS Safewards flier overview and original material developed by Professor Len Bowers, UK (17).

Positive outcomes of Safewards in relation to reducing restrictive practices were reported for the original RCT in the United Kingdom, which showed a significant decrease in conflict events (15%) and containment events (24%) (18). Subsequent evaluation of Safewards in Australia and internationally has reported mixed success. Maguire and colleagues (19) reported high implementation fidelity and fewer conflict events alongside improved ward atmosphere in a forensic mental health ward in Australia. Their study gathered consumer and staff perspectives regarding Safewards during fidelity checks, highlighting positive practice change, enhanced safety and more respectful relationships. A study in Southern Denmark found reductions in coercive measures and forced sedation after the implementation of Safewards, although they were unable to report the fidelity to the Safewards model (20). Several studies have reported low fidelity to the Safewards interventions and challenges in implementing Safewards (21, 22). Researchers have offered a number of possible explanations for this, including lack of management buy-in, lack of training (21), competing priorities in the organization and poor staff attitudes (22). To date, no published research reports the experiences of consumers in acute inpatient mental health wards when Safewards has been successfully implemented.

In light of state and national policies (23, 24) to reduce the use of restrictive interventions and deliver recovery-oriented care in Victorian inpatient mental health settings, the Victorian Government funded implementation of Safewards. Seven self-selected health services implemented Safewards across 18 wards in urban and regional Victoria. Our team was commissioned to conduct an independent evaluation of Safewards in Victoria. The project included evaluating training outcomes, impact of Safewards from consumer and staff perspective, and short-term and long-term outcomes related to implementation fidelity and seclusion rates. Findings have shown that local health service training for ward staff was successful in enhancing the knowledge, confidence, and motivation of staff to implement Safewards (25), and that seclusion rates were significantly reduced by 36% at 12-month follow-up in adult and youth wards implementing Safewards (26).

The development of Safewards has been reliant on nursing literature to date, benefiting very little from the important perspective of consumers. Consumer views were gathered as part of the Victorian evaluation. The aim of this study was to describe the impact of Safewards on consumers’ experiences of inpatient mental health services.

## Methods

### Design

A cross-sectional postintervention survey design was used to study consumer perspectives. Consumers were surveyed between January and March 2016, 9–12 months after Safewards was first implemented, at which time on average 9 of the 10 interventions were implemented. Therefore, regardless of the consumer’s length of stay, they were all exposed to Safewards for most if not all of their admission.

### Setting

This study is based on inpatient mental health wards in both metropolitan and regional Victoria. The average length of stay in acute wards in Victoria is 9.5 days (1). Our study reports data from four of the seven health services that opted to implement Safewards. Four health services agreed to consumers being approached to participate, providing either a consumer consultant or a nurse educator to facilitate the completion of surveys. The inpatient services were adult, adolescent/youth, and aged acute wards and secure extended care units.

### Participants

Current consumers in 10 wards from four health services were invited to take part in the consumer survey. Consumers were approached by either a consumer consultant or nurse educator (who did not have direct consumer contact in the ward). If the consumer was interested to hear more about the study, s/he was given a participant information and consent form.

### Measures

The purpose-designed survey included demographic characteristics and both quantitative and qualitative questions regarding the acceptability, applicability, and impact of the Safewards model and 10 interventions. Five quantitative questions covered: 1) recall of the model and each intervention, possible responses were “yes,” “no,” “unsure”; 2) how worthwhile participants thought Safewards was for them using a 5-point Likert scale: 1 = poor, 2 = fair, 3 = good, 4 = very good, 5 = excellent; 3) how frequently they saw or were involved in the interventions; 4) the impact of Safewards on the atmosphere of the ward; 5) the impact of Safewards on four conflict events, that is, property damage, absconding, physical conflict, and verbal conflict. These conflict events were agreed upon by the researchers and the Government team piloting Safewards as the most relevant in the Victorian context at the time.

A 5-point Likert scale was used to answer the final three questions, whereby 1 = never, 2 = rarely, 3 = sometimes, 4 = usually, 5 = always. Participants who reported the Safewards model or any of the interventions as either “excellent” or “poor” were asked to provide extra information in response to open-ended questions. One text box was available for each response option. The decision to prompt for qualitative responses associated with the two outermost ratings was first pragmatic because we were conscious not to overburden participants. Second, we were keen to elicit the richest consumer views. So, we targeted qualitative follow-on questions to those who had a clear positive or negative view of the issue with prompts, such as if you rated the Safewards model or any of the interventions a “poor” can you briefly describe why the model/interventions were not suitable for your unit?

We next chose to prioritize detailed qualitative feedback regarding the 5 (of the 10) Safewards interventions that are specifically designed to involve consumers.

Further qualitative questions were posed about each of the five interventions, which directly involve consumers, the questions were 1) What do you think of the Clear Mutual Expectations on your unit and were you involved in their development?; 2) What were the Mutual Help Meetings like from your perspective?; 3) What did you think of the Calm Down Box, what was your favorite thing in the box?; 4) Did you feel that the discharge messages were helpful for you and did/will you write your own?

### Procedures

The plain language statement and consent form and the administering consumer consultant or a nurse educator made clear that participation was voluntary and that participants could withdraw at any time. The survey was hosted on SurveyMonkey; participants chose to complete the survey themselves or have the support of a consumer consultant/nurse educator. Ethics approval was obtained *via* the Victorian Human Research Ethics Multi-site process (ID 15225L) for each of the involved services.

### Data Analysis

Quantitative data were analyzed descriptively using SPSS version 22. Weighted averages for the Likert scales were calculated using the number of people who selected a given response and the weighting of that response. Qualitative data were analyzed using a thematic approach guided by the six-step approach outlined by Braun and Clarke (27). We elected to use an inductive process to uncover emerging themes (28). The steps we took were 1) to become familiar with the data, whereby the qualitative comments were read and counted to gain an understanding of the spread of feedback from participants; 2) initial codes were generated about the data, particularly assessing the spread of positive, negative, and neutral comments to provide a sense of the overall perspective of participants about Safewards; 3) comments of three or more words (i.e., those with some meaning to be elucidated) were categorized according to emerging themes; 4) we reviewed and where necessary reorganized the data according to the themes; 5) we discussed the names and definitions of each theme to ensure that they captured the essence of the data. Last 6), the analysis was written up and examined to ensure accurate representation of the data according to the themes. To strengthen the rigor of this analysis, two researchers, JF and BH, conducted steps 1 and 2 of analysis independently before discussing and refining the initial codes and undertaking the remaining steps.

## Results

Although 72 participants started the survey, not all completed every item, so valid participant numbers are presented throughout the results. **Table 2** shows the service type, participants’ demographic characteristics, and length of current admission. Most participants were in adult services, mainly English-speaking, half were female, and on average 40 years of age. For most participants, their current admission had been from 1 to 4 weeks in duration at the time of participation.

**Table 2:** Participant demographics.

	Frequency	%
**Gender**		
Male	29	48
Female	31	52
Other	0	0
**Language**		
English	54	92
Other	5	8
**Aboriginal Torres Strait Islander Status**		
No	58	81
Aboriginal	2	3
Torres Strait Islander	0	0
Both	0	0
Missing	12	17
Age, mean and range	40 years	18–78
**Service type**		
Adult acute	46	64
Adolescent/youth acute	4	6
Aged acute	2	3
Secure extended care	8	11
missing	12	17
**Length of current admission**		
Less than one week	8	11
1–2 weeks	20	28
2–4 weeks	15	21
1–3 months	11	15
More than 3 months	6	8
Missing	10	14

### Use of Safewards: Participants Recall and Perception of Acceptability

**Table 3** demonstrates that participants recalled the interventions to varying degrees. The interventions directly involving consumers were more frequently remembered. **Table 3** also displays the weighted average of responses on the Likert scales and of how worthwhile participants believed each intervention to be, highlighting that participants rated all interventions good to very good with slight variation. More variability was evident in the frequency with which each intervention was used on the ward.

**Table 3:** Participant feedback about each intervention.

Intervention	Recall the use of interventions, *n* = 70			Acceptability and applicability		Frequency of use in the unit	
	Yes (%)	No (%)	Unsure (%)	n	Weighted average	n	Weighted average
**Clear Mutual Expectations**	33	45	22	40	3.33	39	2.67
**Soft Words**	46	30	23	39	3.38	40	2.83
**Talk Down**	29	49	22	36	3.08	33	2.58
**Positive Words**	48	35	17	42	3.43	43	3.09
**Bad News Mitigation**	22	46	32	30	3.43	29	2.48
**Know Each Other**	67	20	13	55	3.4	51	3.25
**Mutual Help Meeting**	81	10	9	61	3.52	56	3.63
**Calm Down Methods**	62	19	19	45	3.4	43	3.21
**Reassurance**	54	25	22	43	3.51	40	3.13
**Discharge Messages**	68	22	10	55	3.24	49	3.02

### Impact of Safewards: Quantitative Data

**Figure 2:**
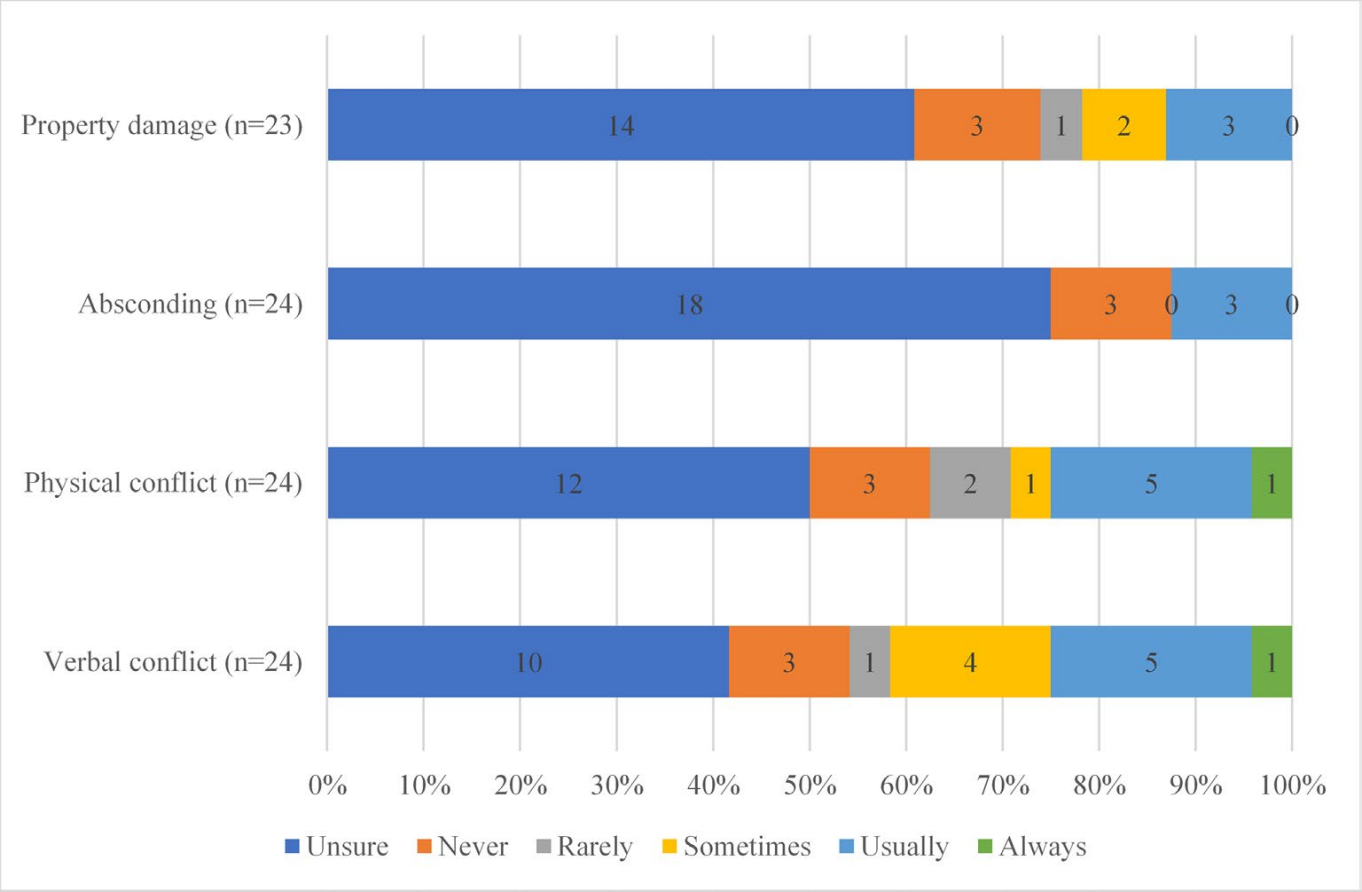
Participants report of the impact of Safewards on conflict events.

**Figure 2** displays participants’ impression of whether four conflict events had reduced in frequency since the introduction of Safewards. Participants were most unsure about absconding and property damage; a small number of participants believed that Safewards never helped or usually helped. Participants were clearer about the impact of Safewards on physical and verbal conflict, with about 25% of participants reporting that Safewards usually or always helped to resolve physical and verbal conflict.

**Figure 3:**
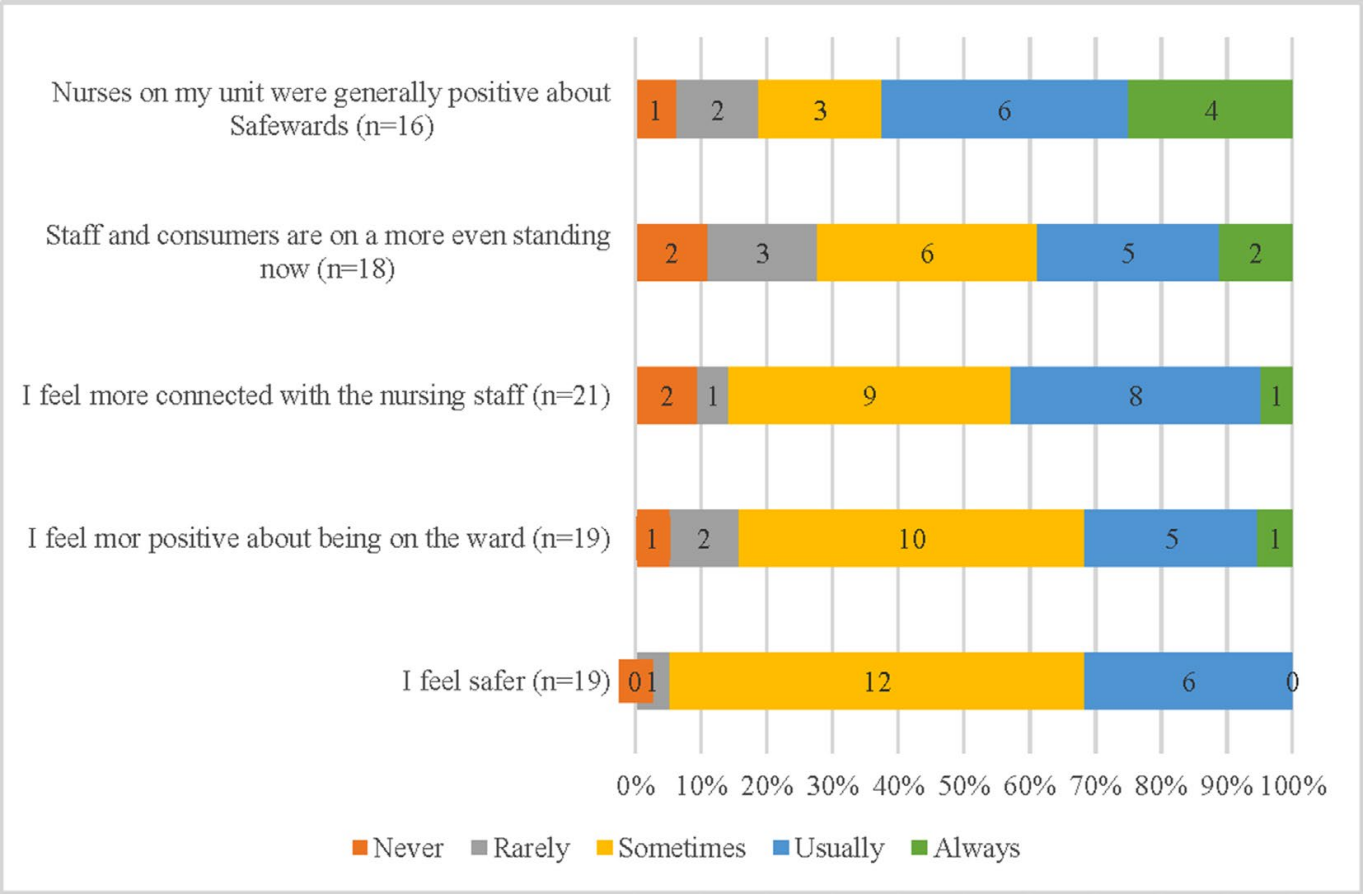
Participants report of the impact of Safewards on the feel of the ward.

**Figure 3** displays five statements about consumer’s experiences of being “on the ward” while Safewards was being implemented. A small number of participants, between 16 and 21, chose to answer these questions. Those who did reported they felt safer in the ward (95% sometimes or usually), more positive about being in the ward, and more connected with the staff (85% sometimes–always). Most participants believed that staff and participants were “on a more even standing” (70% sometimes–always).

### Impact of Safewards: Qualitative Data

The following section provides the results of the thematic analysis of qualitative data shared by participants. First, in 30 instances, participants took up the opportunity to provide open-ended responses regarding why they thought the model or intervention was unsuitable/suitable for their ward, when they had rated either the Safewards model or any of the 10 interventions *poor* (12 participants) or *excellent* (18 participants). Second, participants responded 198 times to open-ended questions about each of the five interventions that directly involve consumers, Mutual Help Meeting (48 comments), Know Each Other (37), Clear Mutual Expectations (36), Calm Down Methods (32), and Discharge Messages (45). **Table 4** displays the overall number of participants who provided their views about each of the interventions and the nature of their comment, positive, negative, or neutral. The following presents a synthesis of qualitative data arranged by six themes that emerged from the data: *Respect, Hope, Sense of community, Safety and sense of calm, Patronizing language and intention*, and *Implementation in practice*.

**Table 4:** Number of participants who provided comments and the general nature of their comments.

	Total number of comments	Positive comments, e.g., Very helpful	Negative comments, e.g., It’s childish	Neutral comments, e.g., I didn’t know about it
**Mutual Help Meeting**	48	37	6	5
**Know Each Other**	37	27	6	4
**Clear Mutual Expectations**	36	24	4	8
**Calm Down Methods**	32	13	1	18
**Discharge Messages**	45	30	1	14

#### Recognition and Respect

The theme of recognition and respect arose from Clear Mutual Expectations and the Mutual Help Meetings, where participants highlighted more fair expectations that showed recognition of personhood and led to increased respect from staff. The use of Clear Mutual Expectations has reportedly resulted in fair expectation and positive changes related to less bullying from staff and comfort in knowing what is expected of consumers and staff “Good to know what’s expected of you, and also staff.” Increased recognition was also felt by participants taking part in Mutual Help Meetings, by enabling consumer voice to be prioritized, as illustrated by the following: “Meetings provide valuable information and provide clients a voice in the running of the facility and ownership.” Involvement in the development of Clear Mutual Expectations was reported by two participants. The concept of mutual respect was highly valued as the following quote illustrates. “Treat people as you would like to be treated, yes I was involved [in the development] and asked for respect.”

#### Hope

*Hope* was a theme that arose from Discharge Messages, and quotes from two participants illustrate this: “If inpatient you’re in a dark place, these bring you back to reality, safe and hope” and “Give people motivation to get better.” Participants saw the messages as “positive” and “helpful”; many reported that they would contribute a message as they were discharged, while others were reluctant to contribute because of being unsure of what to say.

#### Sense of Community

*Sense of community* refers to experiences of improvements in relationships between consumers and other consumers as well as between consumers and staff. A sense of belonging arose from participants being involved in Know Each Other, and responding to the specific agenda items in the Mutual Help Meetings all helped to reduce feelings of social isolation:

“[Mutual Help Meeting] Helpful introduces you to people. Helps improve your stay. Gives your OT a better understanding of how to improve things on the ward. [The round of] Thanks, the people who have done positive things for you.”

The Mutual Help Meetings were received positively by participants who reported that they were “very productive,” “great idea,” and “helpful.” Some participants highlighted increased consumer participation and consumer voice in the day-to-day running of the ward, resulting in an increased *sense of community*, “Very good. Make you feel part of a team. Feel positive.”

Know Each Other was viewed positively by many, for example: “I’ve always wanted this.” Comments provided by participants detail reasons why this intervention was viewed favorably, particularly for increasing the *sense of community* in the ward. “Knowing others helped me communicating with others” and “Beneficial to have that rapport, makes me feel included.” Other participants noted the impact this intervention had upon their view of staff as part of the ward community and that they appreciated knowing a little about the doctors and nurses.” “This is a way of showing that staff are human.”

#### Safety and Sense of Calm

This theme encompasses a change in the general feel of the ward as being a safer and calmer place as well as underscoring individuals’ experiences of feeling calmer through the use of Calm Down Methods. Participants reported on the impact of Safewards as a whole impacting on overall sense of safety and calm personally and among people in the ward, for example: “Keeps everyone calm,” “Useful and helps keep me safe and other patients calm as well,” and “Feel more safer & stronger, it has been very educational.”

Participants engaged with the items from Calm Down Methods as illustrated by the following: “hand cream, spray, shower gel, I have my own box.” Furthermore, participants appreciated the opportunity for self-soothing, with a variety of options to choose from “I really like the dencorub smell it helps me” and “Yeah good, like the weighted blanket, like the light globe.”

Clear Mutual Expectations was also found to facilitate a safer environment as noted by one participant “It’s good, no more bullying.”

#### Patronizing Language and Intention

The theme *patronizing language and intention* draws attention to the notion that some participants felt that not all of the interventions are suitable and respectful of consumers. This theme was evident across three of the five interventions. It encompasses a clear strand of negative consumer experience of Safewards.

Six participants commented that they did not find the Mutual Help Meetings useful either because they didn’t see a positive outcome from the meetings or because they found the concept to be condescending “Don’t like ‘school behaviours’ being incorporated, should be more adult.”

Two participants did not hold a positive view of the Calm Down Methods intervention because of their perception that the language and intention were childish “Inappropriate use of words, e.g., calm down” and “Calm down box—it’s for children. I don’t think it’s respectful to treat people as a child.” One person shared a view of disapproval about the intention of Discharge Messages overall “Discharge Messages are cliché, [I] won’t contribute.”

#### Implementation in Practice

The theme *implementation in practice* reveals that participants observed that implementation and appropriate use of interventions are dependent on staff being willing and involved. One participant shared the insight about Safewards in general that “These [the interventions] were not used by the nurses, medication was offered rather than talking.”

Doubts were also raised by several participants about staff ability to carry out the Clear Mutual Expectations and the variability between different staff, for example, those who are night or part-time staff “Full-time staff are usually better at it than casual/part-time staff, in my experience.” One consumer highlighted lack of staff participation in Know Each Other. “Not all staff participated.” Overall, responses indicate that participants see the value in skilled staff incorporating Clear Mutual Expectations into their practice and building rapport through Know Each Other but note that practice can be inconsistent.

One participant raised concerns about Know Each Other and need for privacy in the ward “Meetings are good because they’re anonymous, not good to have private life being portrayed. A verbal group where this is talked about would be fantastic.”

Many detailed responses regarding Mutual Help Meetings provide evidence that the intervention was being implemented as intended, for example: “Suggestions and requests to make improvements and a time for thanks,” highlighting some of the key agenda items presented in the intervention information.

In the main, the consumer survey responses showed a high level of awareness of the Safewards implementation and nuanced perspectives on its practices and impact. Overall, the qualitative data suggest that many of the participants were providing feedback based on current and previous experience in inpatient settings, for example, reporting less bullying. The majority of participants reported positive views and experiences of most of the interventions, expressing positive changes in relationships between consumers and staff as well as with other consumers. Participants were also positive about having input into the ward environment and being clearer about what is expected of everyone. A smaller number of participants were critical of certain aspects of Safewards, such as Calm Down Methods, or were critical that staff had not implemented some of the interventions adequately.

## Discussion

The aim of this paper was to describe the impact of Safewards on consumers’ experiences of being in an inpatient mental health ward. Sixty to eighty percent of participants recalled the consumer-focused interventions, except for Clear Mutual Expectations. Additionally, some of the practice-based interventions were recalled well, such as Reassurance, Soft Words, and Positive Words. Furthermore, participants were generally positive about the interventions and thought they were being implemented to varying degrees across the 10 wards. This high level of awareness among inpatient consumers of an inpatient model of care is (arguably) not typical (29).

Consumers offered considerable feedback on the experience and overall impact of Safewards. The quantitative findings highlight that some participants were more positive about being in the ward, feeling safer, and more connected with nursing staff as a result of Safewards. In terms of conflict events, participants highlighted a modestly positive view that Safewards interventions were serving to reduce the impact of physical and verbal aggression in the wards.

The qualitative findings of this study provide important context and depth to the quantitative findings. The following sections discuss each of the themes in turn integrating the quantitative and qualitative findings. We have used these areas to highlight, where appropriate, the alignment of these findings with recovery-oriented concepts, which are predominant in the literature reporting inpatient consumers’ expectations and experiences of services.

### Respect

Respect from staff for participants and between the consumers was important in the feedback provided and mirrors the findings of Maguire et al. (19), who reported that both consumers and staff felt increased respect between the two groups (30). Participants in our study reported that Clear Mutual Expectations and the Mutual Help Meetings played a part in increasing the feeling of respect from staff for participants. This finding was further supported in the quantitative data, where some participants reported that staff and consumers were on a more even standing since Safewards implementation. Previous research has highlighted that consumers value being respected by staff, and it has a direct influence on the care they receive (31). More specifically, when feeling better (i.e., reduced symptoms), consumers report valuing increased influence over their care (32). Previous research suggests that consumers feel more respected when they are listened to about their own preferences during care (33). Participants in this study discussed being listened to in the Mutual Help Meeting, in the development of Clear Mutual Expectations, and in the choices they could exercise when using Calm Down Methods.

### Hope

Hope is a core concept in the definition of recovery-oriented practice. In a synthesis of studies on consumer experiences of involuntary treatment, the concept of hope was found to be lacking in many experiences of care but essential to recovery (34). Consumer participants discussed the concept of hope when talking about Discharge Messages, stating that the messages were important in giving them hope, helping them to focus on staying positive. Furthermore, participants reported feeling more hopeful about being in the ward and feeling an increased sense of community because of the Mutual Help Meetings. Often, this was because of meetings increasing participants’ sense of inclusion and agency, also core components of recovery (35).

### Sense of Community

The Mutual Help Meetings contributed to participants feeling more connected with fellow participants; there was appreciation for gaining and providing support to one another and thus feeling safer around one another. Furthermore, a positive sense of community was reported because of Mutual Help Meetings and Know Each Other, which can be related to social inclusion, connection to community, and experiences of citizenship. Hyde and colleagues (35) found that consumers valued such reciprocal support and it reduced feelings of isolation in the ward. Once consumers establish relationships with peers in the ward, they are grateful that, in some instances for the first time and in the midst of experiences of their distress, they feel understood. This finding accords with studies of consumer appreciation of emerging peer support roles in inpatient care (35).

### Safety and Sense of Calm

The literature on safety in acute wards is vast; however, of particular relevance to this study is the literature showing that feelings of safety are enhanced when consumers feel valued, understood, and respected by staff (36, 37). Participants overall reported feeling safer, and participants who rated the model and interventions as excellent reported one of the key changes was that the ward felt calmer, which led to them feeling safer. This finding concurs with reports of consumers from a forensic mental health ward who stated that the ward was calmer and they felt safer after the implementation of Safewards (19).

There are several mechanisms by which the increased sense of safety may have occurred. In times of high acuity, consumers have reported that having predictable services contributed to a feeling of safety (32); in this light, Clear Mutual Expectations was viewed by participants in this study as beneficial. Furthermore, in their critique of Safewards from the perspective of consumers, Kennedy et al. (38) report that Know Each Other could increase consumers’ sense of safety through holding some everyday knowledge about staff.

Evaluation of the Safewards implementation in Victoria in these same wards revealed a reduction in the use of seclusion, which may have impacted on the sense of safety in the ward (26). This notion is supported by converse findings of previous research that consumers feel unsafe in wards where restrictive practices are used by staff to maintain control and gain compliance (33). Previous research has highlighted that key stakeholders—consumers, carers, and staff—consider that restrictive practices are incompatible with recovery-oriented practice (39). So interventions that reduce restriction can be expected to result in an increased sense of safety.

### Patronizing Language and Intention

Concerns voiced by some participants suggest that some components of Safewards can be viewed as patronizing, with such comments about Calm Down Methods being more suited for children. Language is particularly powerful with potential to reinforce condescending views of mental illness (40). This challenge can be addressed in the first instance by changing language used for interventions, such as Calm Down Methods, and by providing consumers with the opportunity to choose the tools available for this intervention. There is considerable scope for consumer perspectives to be foregrounded using coproduction processes, when Safewards interventions are reworked and new interventions are developed (38). Furthermore, the astute consumer critique of staff language and intent makes clear the need to ensure that the implementation of Safewards and other interventions is not undermined by a superficial approach that misses the intent of an intervention or strays from the model and underpinning evidence (41, 42). In large organizations, it is possible that some staff miss the meaning of such a program or that an ethos is not sustained after the initial burst of training.

Also, there is scope, based on this evidence and other consumer expert contributions (38), to refine specific language in the interventions, including changes to “Calm down.” Steps have been taken already in different local settings such as “Chill kit” in some adolescent units.

### Implementation in Practice

A small number of participants highlighted their observation that staff did not implement all of the interventions, and low implementation fidelity has been reported in other studies (21, 22). Qualitative data regarding each of the five consumer-engaged interventions illustrated that participants had a clear understanding of the Safewards interventions and their intent, which suggests that they had experienced the interventions as they were intended. This finding supports previous reports that fidelity to the interventions was high (on average, wards were implementing 9 or 10 of the interventions) at the 9- to 12-month time point after the initial trial of Safewards finished (26). It is, therefore, also likely that the five interventions that are less visible to participants—Reassurance, Positive Words, Soft Words, Talk Down, and Bad News Mitigation—played a part in the reported general experience of the wards as calmer and safer. In addition, the appropriate implementation of Positive Words and Soft Words is likely to contribute to participants’ perceptions of respect from staff members. The implementation of Reassurance, Talk Down, and Bad News Mitigation is likely to have impacted on the sense of calm in the ward and the resulting feeling of safety.

### Limitations and Strengths

There are three key limitations in the present study. First, the data were not representative of all wards involved in the trial because not all services granted ethics approval for consumers in inpatient units to be recruited and surveyed. Second, completion of surveys was variable; most participants were more inclined to provide qualitative comments than to answer the quantitative questions about flashpoints and impact of Safewards on the environment. It is impossible to know why fewer participants chose to answer the quantitative questions. Nevertheless, the qualitative and quantitative survey findings align well. Third, the sample may have been skewed toward those who were at that moment well enough to complete surveys and/or those who had a more positive experience of Safewards. Nonetheless, the participants were knowledgeable about Safewards and able to give rich responses.

Notwithstanding the limitations, our paper has a number of strengths. First, our research gives priority to the consumer voice across adolescent, adult, and aged acute inpatient wards and secure extended care units. Hence, it is one of few papers to consider the views of consumers about Safewards, and it highlights that Safewards can be well received in mental health services beyond the adult acute wards for which it was designed. Second, a strength of the study was that most participants had been present in the ward for at least 1 week and even up to 3 months, with ample opportunity to be involved in Safewards and experience the difference it made. Third, the timing of the survey was a strength to this research because Safewards was well implemented, thus ensuring consumers had good exposure to the interventions as they were intended.

## Conclusions

Most participants were positive about Safewards, highlighting important improvements in their experiences of inpatient care since implementation. The findings of the present study highlight that Safewards offers a pathway to improving the relationship between consumers and staff and enables a move toward recovery-oriented practice. Qualitative comments from consumer participants have begun to elucidate findings in previous research, particularly regarding how and why some of the Safewards interventions alleviate negative experiences of consumers. Furthermore, the key themes arising from the qualitative data highlight the alignment between the impact of Safewards interventions and recovery-oriented practice, which is highly valued by consumers.

Safewards is making a difference to consumer experiences on psychiatric inpatient wards. However, Safewards needs ongoing attention to remain relevant. The consumer voice was largely missing from the initial development of the interventions (although consumers were consulted in selecting which interventions to trial) and the strong reliance on published literature for the development of the model and interventions may mean that Safewards is backward looking. To keep Safewards relevant, we now need to engage with the current day and critical perspective from consumers, to codesign ongoing development and evolution of the interventions based on evaluation findings. To this end, we look to suggestions made by Kennedy et al. (38) to extend Safewards to include varied interventions from the original 30, to maintain the momentum of change.

In the current context, with increasing importance being placed on coproduction and consumer perspectives as central to improving service delivery, we must rely on new ways to engage with the critical consumer perspective. This is especially important regarding promising models such as Safewards that were developed using literature that existed before the imperative “nothing about us without us” (43), the rights-oriented call arising from the mental health consumer movement. The credibility of the next stage of Safewards development rests on greater consumer voice at the level of collaboration, consumer-preferred language, and intervention refinements.

## Ethics Statement

This study was conducted in accordance with and after recommendations from Victorian Human Research Ethics Multi-site process (ID 15225L). Participants were provided a Plain Language Statement and Consent Form. Participants had the opportunity to ask questions before signing the consent form. Completion of online surveys was anonymous. The protocol was approved by the Monash Health Human Research Ethics Committee.

## Author Contributions

JF and BH were involved in the development of the study, data collection, and analysis. JF, SB-H, BH and LB were involved in the interpretation of data. JF, SB-H, BH, SK and LB were involved in the writing and editing of the manuscript.

## Funding

This paper forms part of the work towards a PhD which is supported through an Australian Government Research Training Program Scholarship. JF is supported by NHMRC PhD Research Scholarship 1133627. SK is supported by NHMRC Research Fellowship APP1078168. The Department of Health and Human Services, Government of Victoria funds clinical services across the state. This independent evaluation was financially supported by the Office of the Chief Mental Health Nurse, in the Department of Health and Human Services, Government of Victoria.

## Conflict of Interest Statement

The authors declare that the research was conducted in the absence of any commercial or financial relationships that could be construed as a potential conflict of interest.
